# Prevalence and risk factors for cervical cancer and pre-cancerous lesions in Rwanda

**DOI:** 10.11604/pamj.2015.22.26.7116

**Published:** 2015-09-11

**Authors:** Jean Damascène Makuza, Sabin Nsanzimana, Marie Aimee Muhimpundu, Lydia Eleanor Pace, Joseph Ntaganira, David James Riedel

**Affiliations:** 1 Rwanda Biomedical Center, Kigali, Rwanda; 2 Brigham and Women's Hospital, Boston, MA, USA; 3 University of Rwanda, College of Medicine and Other Health Sciences, School of Public Health, Rwanda; 4 Institute of Human Virology and Division of Infectious Diseases, University of Maryland School of Medicine, Baltimore, Maryland, USA

**Keywords:** Rwanda, cervical cancer, screening, VIA

## Abstract

**Introduction:**

Cervical cancer prevalence in Rwanda has not been well-described. Visual inspection with acetic acid or Lugol solution has been shown to be effective for cervical cancer screening in low resource settings. The aim of the study is to understand the prevalence and risk factors for cervical cancer and pre- cancerous lesions among Rwandan women between 30 and 50 old undergoing screening.

**Methods:**

This cross-sectional analytical study was done in 3 districts of Rwanda from October 2010 to June 2013. Women aged 30 to 50 years screened for cervical cancer by trained doctors, nurses and midwives. Prevalence of pre-cancerous and cancerous cervical lesions was determined. Bivariate and multivariate logistic regressions were used to assess risk factors associated with cervical cancer.

**Results:**

The prevalence of pre-cancer and invasive cervical cancer was 5.9% (95% CI 4.5, 7.5) and 1.7% (95% CI 0.9, 2.5), respectively. Risk factors associated with cervical cancer in multivariate analysis included initiation of sexual activity at less than 20 years (OR=1.75; 95% CI=(1.01, 3.03); being unmarried (single, divorced and widowed) (OR=3.29; 95% CI=( 1.26, 8.60)); Older age of participants (OR= 0.52; 95% CI= (0.28, 0.97)), older age at the first pregnancy (OR=2.10; 95% CI=(1.20, 3.67) and higher number of children born (OR=0.42; 95%CI =(0.23, 0.76)) were protective.

**Conclusion:**

Cervical cancer continues to be a public health problem in Rwanda, but screening using VIA is practical and feasible even in rural settings.

## Introduction

Cervical cancer is the fourth most common cancer in women worldwide and the second most common female cancer in women aged 15-44 years old worldwide [1]. In 2012 there were an estimated 528,000 new cases of cervical cancer and 266,000 deaths from cervical cancer, with 70% of those deaths occurring in developing countries [2]. In Sub-Saharan Africa, cervical cancer accounts for 22.5% of all cancer cases in women, and the majority of women who develop cervical cancer live in rural areas [3]. Eastern Africa is one of the most heavily affected areas with an incidence of more than 30 cases per 100,000 women per year [4]. Rwanda has a population of 11 million with 2.72 million women aged 15 years and older who are at risk of developing cervical cancer [5]. Current estimates indicate that every year nearly one thousand women are diagnosed with cervical cancer and almost 700 die from the disease [6]. Cervical cancer ranks as the most frequent cancer among women in Rwanda, and the most frequent cancer among women between 15 and 44 years of age [7]. The estimated incidence of cervical cancer in Rwanda is 49 cases per 100,000 women per year, much higher than the estimated rates in Eastern Africa and worldwide, 34.5 and 16 new cases per 100,000 women, respectively [8]. Several key risk factors for cervical cancer are common in sub-Saharan countries, including prolonged HPV infections and HIV/AIDS which is endemic in this region (UNAIDS report 2012). Other risk factors include debut of sexual activity before age of 20 years old, multiple sexual partners, tobacco smoking, oral contraceptive pill use for more than 5 years, history of cervical cancer in the family, high parity (more than 3 children born), and immune-depression due to malnutrition or other systemic diseases [9].

In low resource countries, cytology-based screening programs and/or DNA typing of HPV are usually beyond the capacity of many health services. Visual inspection of the cervix using acetic acid (VIA) or Lugol's iodine (VILI) to highlight precancerous lesions allows identification of pre-cancerous lesions in the clinic instead of the laboratory. With adequate training, any health care provider, including doctors, nurses, or nurse-midwives, can effectively perform the procedure [10]. VIA may perform as well as or better than cervical cytology in identifying pre-cancerous lesions [10, 11]. Different studies have demonstrated that using VIA, trained physicians and other providers correctly identified between 45% and 79% of women at high risk of developing cervical cancer [10]. In comparison, the sensitivity of cytology has been shown to be between 47 and 62% [12]. Paired with cryotherapy, VIA has successfully been implemented as a relatively simple, acceptable, and cost effective method of treating cervical lesions and preventing development of cervical cancer in resource-limited settings [13–17]. In 2010, the Government of Rwanda initiated training for health providers in VIA and cryotherapy, with the goal of launching a national cervical cancer screening program for all women between 30 and 50 years old. In combination with trainings, initial screenings were conducted in several districts to collect baseline information. Having accurate data on cervical cancer prevalence will help guide control and prevention of cervical cancer in Rwanda as it can help determine the effectiveness of screening and treatment programs and guide resource allocation for treatment. This study was conducted to further understand the prevalence and risk factors for cervical cancer and pre- cancerous lesions among Rwandan women between 30 and 50 old undergoing VIA screening in 3 districts.

## Methods

*Type of study*: this is a descriptive cross-sectional and analytical study. Data collection was done from subjects at the time of cancer screening through use of a standardized questionnaire.

*Study population*: the study population consisted of women aged 30 to 50 years old who were screened for cervical cancer in 3 districts in Rwanda from October 2010 to June 2013. Sensitization on cervical cancer screening was done by radio advertisements and with the help of community health workers before and during the screening period. Participants were recruited from the population of the 3 districts without regard to HIV status. Women were excluded from the study if they were more than 20 weeks pregnant; less than 12 weeks post-partum; had a previous history of treatment of cancerous lesions; had a known allergy to acetic acid; or if they had undergone total hysterectomy. By convenience this study was conducted in 3 districts offering screening during the early stages of the national campaign: Musanze District (with 2 sites: Ruhengeri district hospital and Kinigi health center) from North Province; Rwamagana (two sites: Rwamagana District hospital and Avega health center); and Kayonza district (five sites: Rwinkwavu and Gahini district hospitals, Mukarange, Nyamirama and Kabarondo health centers) from Eastern Province.

*Data collection procedures*: a chart abstraction instrument was elaborated for collecting data from participant files. Information in files was collected by trained providers during systematic screening, and data were extracted retrospectively from those files stored in the three sites by providers from the respective sites.

*Screening procedures*: screening was done by medical doctors, nurses, and midwives with 3 years of experience in maternity service who had been trained for 2 weeks in theory and practice in cervical cancer screening. The following procedures were followed for screening: (1) Speculum examination: If a lesion suspicious for invasive cervical cancer was noted, colposcopy was performed and women were referred to referral hospitals (Kigali and Butare University Teaching hospitals and king Faycal Hospital) for biopsy. If no lesions concerning for invasive cervical cancer were seen, VIA was performed to detect pre-cancer lesions (acido-white lesions); (2) For VIA positive women with minor lesions, cryotherapy was performed to remove pre-cancer lesions. For VIA positive women with larger lesions, the Loop Excision Electrical Procedure (LEEP) was performed by trained medical doctors; (3) Women with pre-cancer lesions treated with cryotherapy were recommended to follow up after 3 months for clinical evaluation and one year later for re-screening.

*Variables*: two dependent variables of interest were chosen: Invasive cervical cancer was defined as a binary variable with an outcome of diagnosis of invasive cervical cancer. Invasive cervical cancer lesions were diagnosed clinically if lesions suggestive of invasive cancer were found on the cervix. All cases were confirmed by colposcopy and biopsy; cervical pre-cancer was defined as a binary variable. Cervical pre-cancer lesions were diagnosed clinically after application of acetic acid or vinegar at 5%. Independent variables included: socio-demographic variables: these included age, sex, educational level, provenance, marital status and socio-economic status. Socioeconomic status was defined in accordance with the Demographic and Health Survey 2010 [5] considering housing conditions with having access to electricity and improved water and also considering ownership of goods (radio, mobile phone, bicycle, car, agriculture land etc.). HIV status was obtained by self-report; clinical and behavioral variables: age of first intercourse, number of sexual partners, sexual transmitted infections (STIs) were assessed by clinical examinations. For STIs, a syndromic approach was used in diagnosis and treatment, Number of pregnancies and number of children delivered, age of the first and last pregnancy, use of oral contraceptive pills as family planning, HIV status, smoking and alcohol use were considered as independent variables

*Statistical analysis*: double data entry was done with Epi Data 3.1 by trained staff. After data cleaning, data were exported into SPSS 16.0 for analysis. For descriptive and data summary purposes, univariate techniques were applied to single sets of data. Graphical and tabular techniques included frequencies and bar graphs. Summary statistics such as mean and standard deviation were calculated for continuous variables. Bivariate methods were used to show the relationship between variables; contingency tables were used to describe relationship between categorical variables. Tabular methods of describing the relationship between two nominal variables by finding proportions were also employed. Multivariate analysis was used to determine the socio-demographic, behavioral and clinical factors associated with cervical cancer and pre-cancer lesions. Binary and multinomial logistic regressions were used to show the association of any cervical cancerous lesion with different factors In order to facilitate regressions, most variables were made into binary variables (age group, marital status, education level, social economic status, number of pregnancies, number of children born, age of the first pregnancy and number of sexual partners) to facilitate analysis of the association of cervical cancer and pre-cancer with different factors. After bivariate analysis, a series of multivariate regressions were conducted: all variables with P-value below 0.10 in bivariate analyses were included in multivariate regression, after those variables with p-value <0.10 with cofounding variables were also included in another series of multivariate regression to find out final variables associated with any cervical cancerous lesion.

*Ethical considerations*: the Rwanda School of Public Health Internal Review Board approved the study, and site authorizations were obtained from the Ministry of Health for hosting sites.

## Results

### Study population

Socio-demographic characteristics of our study population are shown in Table 1. The mean age of women was 37.0 years with the majority of participants (75%) between 30 and 40 years old. The majority were married (84%), and 81.9% had received no education or primary school only. The majority of women (66.4%) were from the Eastern province. Gynecological-obstetrical and other clinical characteristics among women screened for cervical cancer are displayed in Table 2. Most women reported 4 or more pregnancies (65%). The majority of participants (80%) who were taking oral contraceptives used them for less than 5 years. Half of the respondents had their sexual debut when they were over 20 years old, and two-thirds reported only one lifetime sexual partner. Almost 10% of the population reported a diagnosis of HIV, while nearly 80% reported themselves to be HIV negative; however, 12% did not know their HIV status.

**Table 1:** Socio-demographic characteristics of women screened for cervical cancer

Characteristic	n (%)
**Age** (N=1002)	
30-35 years	478 (47.7)
36-40 years	275 (27.4)
41-45 years	135 (13.5)
46-50 years	114 (11.4)
**Marital status** (N=892)	
Single	16 (1.8)
Married	748 (83.9)
Divorced	66 (7.4)
Widowed	62 (7.0)
**Education completed** (N=895)	
None	187 (20.9)
Primary	546 (61.0)
Secondary	133 (14.9)
University	29 (3.2)
**Socio-economic status** (N=891)	
Low level	309 (34.7)
Middle level	573 (64.3)
High level	9 (1.0)
**District of origin** (N=1002)	
Musanze (North)	206 (20.6)
Rwamagana (East)	131 (13.1)
Kayonza (East)	665 (66.4)

**Table 2:** Gynecological-obstetrical, clinical and behavioral characteristics of women screened for cervical cancer

Characteristic	n (%)
**Number of pregnancies** (N=960)	
1	71 (7.4)
2-3	265 (27.6)
4-5	307 (32.0)
> 5	317 (33.0)
**Number of children born** (N=957)	
1	73 (7.6)
2-3	304 31.8)
4-5	319 (33.3)
> 5	261 27.3)
**Age of the first pregnancy** (N=806)	
< 20 years	276 (34.2)
20-35 years	527 (65.4)
> 35 years	3 (0.4)
**Oral contraceptive pills** (N=102)	
≤5 years	82 (80.4)
>5 years	20 (19.6)
**Age of first intercourse** (N=851)	
<20 years	406 (47.8)
≥20 years	445 (52.3)
**Number of sexual partners** (N=929)	
1	612 (65.9)
2-5	292 (31.4)
>5	25 (2.7)
**Self-Reported HIV Status (N=1002)**	
Negative	787 (78.5)
Positive	98 (9.8)
Unknown	117 (11.7)
**Suspicion of STI (N=1002)**	
No	851 (84.9)
Yes	151 (15.1)

### Cervical pre-cancer lesions (VIA positive) and cancer prevalence

Table 3 shows the prevalence of pre-cancer (VIA positive) and cancer lesions by risk factor. The overall prevalence of cervical pre-cancer lesions was 5.9%, while the overall prevalence of cervical cancer was 1.7% (17 cases out of 1002 women screened). Pre-cancer lesions (VIA positive) were most prevalent in the 30-35 year old group, in those who were married, in the group with no education, in those with low level socio-economic status, in participants with one pregnancy, in participants with one child born, in participants who had the first pregnancy before 20 years old, in participants who had first sexual intercourse before age 20, in those who self-reported HIV positivity, in participants who had had more than 5 sexual partners, and in participants who smoked.

**Table 3:** Prevalence of pre-cancer lesions (VIA positive) and invasive cervical cancer according to different characteristics

Characteristic	N	Number with pre-cancer (VIA positive)	Cervical pre-cancer (VIA positive) prevalence (%)	95% CI	Number with cancer	Cervical cancer prevalence (%)	95% CI
**Total**	1002	60	5.9	4.5-7.5	17	1.7	0.9-2.5
**Age group**							
30-35 years	478	41	8.6	6.1-11.1	4	0.8	0.2-1.7
36-40 years	274	8	2.9	0.9- 4.9	3	1.1	0.1-2.3
41-45 years	135	5	3.7	0.5- 6.9	5	3.7	0.5-6.9
45-50 years	114	6	5.3	1.1- 9.4	5	4.4	0.6-8.2
**Marital status**							
Single	16	0	0	0-0.2+	0	0	0-0.2+
Married	747	50	6.7	4.9-8.5	15	2	1.0-3.0
Divorced	66	3	4.5	0.6-10.0	2	3.0	1.3-7.5
Widowed	62	0	0	0-0.1+	0	0	0-0.1+
**Age of first intercourse**							
<20 years	406	31	7.6	4.8-9.9	7	1.7	0.4-3.0
≥20 years	445	20	4.5	2.6-6.4	8	1.8	0.6-3.0
**Number of sexual partners in lifetime**							
1	612	32	5.2	3.3-6.8	9	1.5	0.5-2.4
2-5	292	18	6.1	3.4-8.9	7	2.4	0.6-4.2
>5	25	4	16.0	0.6-31.4	0	0	0-0.1+

+ When prevalence was 0, 95% CI was calculated according to Wilson test

### Risk factors for cervical cancer lesions

Table 4 shows the characteristics that were risk factors for invasive cervical cancer using bivariate and multivariable analysis. Using binary logistic regression to assess the relationship between cervical pre-cancer or invasive cancer and the independent variables collected, we found that being unmarried was the only risk factor associated with cervical cancer (P= .040 with OR=2.6 with 95%CI= (1.05-6.69)). After bivariate analysis, variables with P<.10 and other variables qualified as confounders for cervical cancer risk factors were included in multivariate logistic regression models to determine their association with invasive cervical cancer or pre-cancerous lesions analyzed by association with cervical cancer lesions (invasive and pre-cancer lesions both) as dependent variables by series (two times) after elimination of variables with P> .05 (multivariate analysis). With multivariate regression, the risk of developing any cervical cancerous lesion decreased with increasing party (OR=0.42; 95%CI= 0.23-0.76). Being older than 40 decreased risk (OR=0.52; 95%CI= 0.28-0.97). The risk increased if age of the first sexual intercourse was less than 20 years old (OR=1.75; 95% CI=(1.01-3.03)); it increased also in participants who live alone (single, divorced and widowed) (OR=3.29; 95%CI=(1.26-8.60)). The risk was increased with age of the first pregnancy less than 20 years old (OR=2.10; 95% CI=(1.20-3.67)). The total number of pregnancies was not associated with cervical cancer.

**Table 4:** Risk factors of cervical cancer and cervical pre-cancerous lesions (VIA positive) among women screened

		Bivariate			Multivariate		
Risk factors	Cervical cancer risk (cancer and pre-cancer lesions) prevalence (%)	OR	95% CI	P	OR	95% CI	P
**Age group**							
30-40 years	7.4	1.00			1.00		
41-50 years	8.4	1.59	0.95-2.66	78	**0.52**	**0.28-0.97**	**040**
**Marital status**							
Married	8.69	1.00			1.00		
Single, widowed or divorced	3.4	2.65	1.05-6.69	**40**	**3.29**	**1.26-8.60**	**015**
**Number of children born**							
1-3 children	9.5	1.00			1.00		
>3 children	6.72	0.68	0.43-1.10	14	**0.42**	**0.23-0.76**	**004**
**Age of the first pregnancy**							
<20 years	9.9	1.59	0.950-2.66	78	**2.10**	**1.21-3.67**	**009**
≥20 years	6.4	1.00			1.00		
**Age of first intercourse**							
<20 years	9.1	1.17	0.59-2.31	310	**1.75**	**1.01-3.03**	**047**
>20 years	6.3	1.00			1.00		
**Number of sexual partners**							-
1 partner	6.7	1.00			-		
>1 partner	6.3	1.40	0.85-2.30	182	-	-	

## Discussion

This study is the first large-scale analysis of population-based cervical screening results using VIA from women aged 30 to 50 years old in Rwanda. The prevalence of cervical cancer and pre-cancer lesions was 1.7% and 5.9%, respectively. Younger age, earlier age of the first pregnancy, early sexual debut, and higher number of children born were all risk factors for cervical cancer. Risk factors for cervical cancer in this study were similar to other studies in sub-Saharan Africa where cervical cancer screening using VIA was used.

This study showed a prevalence of 1.7% for cervical cancer and 5.9% for pre-cancer among our study population. These results are similar to those from other resource limited settings in sub-Saharan Africa; for example, in Nigeria the prevalence of pre-cancer lesions were between 4.8-14% [18]. Compared with a pooled analysis of data from Malawi, Madagascar, Nigeria, Uganda, Tanzania, and Zambia, the prevalence of invasive cancer in the Rwandan setting was similar, whereas the prevalence of pre-cancer in this study was lower. In those six countries, a total of 19,579 clients were screened with 10.1% having positive VIA results and 1.7% with probable cancer [14]. In a study done in northern Thailand, the VIA test-positive rate was 13.3% in nearly 6,000 women, much higher than the prevalence of pre-cancer in our study [19]. The mean age of the women presenting for screening in this study was 37.0 years and most participants were between 30 and 35 years old (47.7%). These findings demonstrate higher participation in screening of younger women, similar to other studies in Africa like in Nigeria, where the population screened was younger than the general population [18]. Older age of participants was also associated with lower risk for invasive and precancerous lesions in adjusted analyses. Because we considered both invasive cancer and pre-cancer lesions together as our outcome of interest in multivariate analyses, the prevalence of pre-cancer lesions affected this association. Another study done in Rwandan women in Kigali that assessed HPV infection and cervical cytology in HIV positive and HIV negative women showed that HPV prevalence and carcinogenic HPV decreased with age [7]. Cervical cancer has long been associated with HIV infection, and in fact, it was added as and AIDS-defining illness to early AIDS case definitions by the CDC. In the present study, women with HIV had a higher frequency of cervical pre-cancer lesions (8.2%), although the association was not statistically significant. As women with HIV have a higher frequency of HPV co-infection, rates of pre-cancer and cancer are typically higher. Previous studies in sub-Saharan Africa have demonstrated a consistent association of cervical cancer and HIV (e.g. Senegal [20]; South Africa, Zimbabwe [21]; and also in other low-and middle income countries [22]. Given the low prevalence of cervical cancer in this study, we were not able to detect an association with HIV infection status.

Similar to prior studies, in these Rwandan women the risk of cervical cancer increased with young age of the first sexual intercourse (OR=1.75; 95% CI=(1.01-3.02) and in women who live alone (single, divorced and widowed) (OR=3.29; 95% CI=(1.26-8.60)). Having sex at earlier age and having multiple partners increases the risk of infection with a high risk human papillomavirus (HPV) [23]. This association was also seen in a case-control study of cervical cancer risk factors in Indian women where maximum risk of cervical cancer was increased in women with a sexual debut at <12 years old (OR=3.5) and also increased in women who had extramarital sexual relationships (OR=5.5) [24]. This association of cervical cancer with sexual behavior also was shown in a case- control study done in Manchester, England where the number of sexual partners was the only risk factor (OR for six or more=3.89) [25]. We linked marital status and number of partners because it is known that living situation is correlated to having multiple sexual partners. VIA was used as the screening test for all study participants. American Cancer Society guidelines suggest cervical cancer screening from 21 years old to 65 years old when using only Pap smear or visual inspection and from 30 years old to 65 years old when HPV DNA test is associated to these above mentioned tests [23]. Because VIA needs to be performed at regular frequency (every year for PLWH and every three years for HIV negative women), the Rwanda MOH has planned to include HPV DNA testing with VIA in the future; HPV DNA test was not yet available in Rwanda at the time of our study.

This study had several limitations. As the study was a convenience sample of women presenting for care from 3 districts in Rwanda, the prevalence results may not be generalizable to the whole of Rwanda or to the East African female population, where HIV infection rates are quite varied. There is also a possibility of selection bias, as women who participated in the screening program may have been more likely to have current symptoms or be more likely to be HIV-infected since the reported prevalence of HIV in the study population was higher than the national prevalence for HIV (10% vs. 3%). The main purpose of this study was to serve as a baseline for further studies and to help in planning for cervical cancer screening in Rwanda; future studies using randomized sampling techniques may give a more complete estimation of the national prevalence. Additionally, this study relied on chart review to confirm clinical variables (e.g. HIV status) which may have led to misclassification based on incomplete or inaccurate documentation. Lastly, there were limited variables available for analysis in the medical records, limiting the number of risk factors and confounders that we were able to study.

## Conclusion

Cervical cancer continues to be a public health problem in Rwanda, but screening using VIA is practical and feasible even in rural settings. These preliminary results from 3 districts should be expanded to develop a complete national picture for Rwanda. Risk factors for cervical cancer in this study (age of participants, age of the first pregnancy, sexual behavior, and number of children) were similar to those previously described. Implementation of cervical cancer screening in all eligible Rwandan women should be a national priority.
